# Supplementary material for: Conserved Control Path in Multilayer Networks
Source: Entropy (Basel). 2022 Jul 15;24(7):979. doi: 10.3390/e24070979 (PMC9324794; doi:10.3390/e24070979)
Supplement: Supplementary file 1 [file entropy-24-00979-s001.zip › Supplementary Material.pdf]

Supplementary Material

Supplementary Notes

A. Process of generating a multilayer SF network

A.1 Generate a random SF network

(1) Start from N disconnected nodes represented by $V=\{v_i,\ i=(1,\ 2,\ ...,\ N)\}$.

(2) Convert node set V into $V^{out}=\{v_i^{out},\ i=(1,\ 2,\ ...,\ N)\}$ and $V^{in}=\{v_i^{in},\ i=(1,\ 2,\ ...,\ N)\}$.

(3) Assign a node weight $w_i^{out,in}=i^{-\alpha_{out,in}}$, $\alpha_{out,in}\in[0,1]$, to each node in V^{out} and V^{in}.

(4) Randomly select two nodes v_i^{out} and v_j^{in} from V^{out} and V^{in} with probability w_i^{out} and w_j^{in}, respectively.

(5) Connect v_i^{out} and v_j^{in} if there is no connection between them, which corresponds to a directed edge from v_i^{out} to v_j^{in}; otherwise, return to step (4).

(6) Repeat steps (4) and (5) until the number of edges reaches the specified value.

(7) Randomly generate a group of edge weights for each edge, where the value of each edge weight ranges from 1 to the number of edges in the network.

(8) The degree distribution under this construction is $P_{out,in}(k)\sim k^{-(1+1/\alpha_{out,in})}=k^{-\gamma_{out,in}}$. Randomly generate a number 0.713 as $\alpha_{out,in}$, which corresponds to $\gamma_{out,in}=2.402$, which allows the degree distribution of the synthetic network to be closely similar to that of most real networks [1].

A.2 Generate an L-layer SF network with different nodes

We are not limited to an L-layer network that has the same nodes among different layers. However, detecting CCPs for a multilayer network in which each layer has distinct nodes is meaningless. Therefore, to ensure that the number of nodes between layers does not vary too much to allow the detection of CCPs, we define

$$differFrac=(N_{\max}-N_{\min})/N_{\min}, \tag{A.1}$$

where $N_{\max}$ and $N_{\min}$ are the number of layers with the most and least number of

nodes, respectively. We randomly generate the number of nodes in the other L–2 layers between $N_{\min}$ and $N_{\max}$. We set $N_{\min}$ = 500 for every synthetic multilayer network. We set $N_{\max}$=575, and *differFrac* is 0.15 for networks we generate to use in Figs. 2(a) and (e) for our experiments.

A.3 Generate an L-layer SF network with common edges

First, we generate a random SF network G_1 with $N_{\min}$ nodes as the first layer of an L-layer network according to the above methods. Second, we randomly select some edges from G_1 with a given proportion of common edges, and consider them as common edges across all L layers in the L-layer network. For the other $L-1$ layers, we retain the common edges and randomly generate the other edges. We set the proportion of common edges for the networks we generate to use in Figs. 2(d) and (e) to 0.5.

A.4 Generate an L-layer SF network with a stable number of different nodes between adjacent layers

We generate a network G_1 with $N_{\min}$ nodes. The number of nodes in the other $L-1$ layers increases uniformly. The incremental number depends on the ratio of different nodes; that is, $N_j - N_i = N_{\min} \times \mathit{propotion}\ \left(j = i+1,\ 1 \le i \le L-1\right)$, where N_i is the number of nodes in the i-th layer of the L-layer network.

We fix every network density to 0.005 for the networks we generate to use in Figs. 2(a) and (d). Additionally, all the networks we generate are weighted. Therefore, to test the robustness of CCPs, we add four types of Gaussian noise (μ=0, σ=1, 3, 5, 25) to the weights of edges in each network (network used in Figs. 2(b) and (d)). We obtain the Gaussian noise generated for every edge using the random.gauss (μ, σ) function [2] in Python, where μ is the mean and σ is the standard deviation.

B. Construct a cancer-related activated signaling network

B.1 Human signaling network

To construct a cancer-related activated signaling network, we first construct a background network, which we call the human signaling network. First, we collect the data of five signaling relationships and corresponding human proteins from the Pathway Commons database [3], which integrates publicly available biological pathways from multiple organisms. The five signaling relationships are controls-state-change-of, controls-transport-of, controls-phosphorylation-of, controls-expression-of, and catalysis-precedes. Then, we treat proteins as nodes and signaling relationships as

directed edges to construct the human signaling network. Moreover, we delete multiple edges between nodes, and finally, there are 148,721 edges between 7,795 nodes, which constitute the human signaling network.

B.2 The Cancer Genome Atlas (TCGA) multi-omics data

We collect the publicly available somatic mutation and expression data of 16 common cancers, where the sample size of the controls of each cancer needs to be at least 10 from TCGA multi-omics data [4]. We use an R packet edgeR [5] for differential expression analysis and obtain *p*-values for all genes. The 16 cancers are BLCA, BRCA, COAD, ESCA, HNSC, KICH, KIRC, KIRP, LIHC, LUAD, LUSC, PRAD, READ, STAD, THCA, and UCEC.

B.3 Cancer-related activated signaling network

We construct a cancer-related activated signaling network based on the human signaling network and TCGA multi-omics data. In previous studies, researchers indicated that significantly co-expressed genes are inclined to interact (i.e., to produce connection edges in the network) [6,7]. Therefore, we not only consider the expression of a single gene but also co-expressed gene pairs of cancer-related data from TCGA. We use the method used by Liu et al. [8] to calculate the activity of edges, as in the following formula:

$$activity\left(e\left(\langle g_1, g_2\rangle\right)\right) = f\left(diff\left(g_1\right), diff\left(g_2\right), corr\left(g_1, g_2\right)\right), \tag{B.1}$$

where $e\left(\langle g_1, g_2\rangle\right)$ represents the directed edge from gene g_1 to gene g_2, $diff\left(g_1\right)$ and $diff\left(g_2\right)$ are *p*-values that indicate the significance of the differential expression (*p*-value) of genes g_1 and g_2 in cancer samples compared with control samples, $corr\left(g_1, g_2\right)$ is the co-expression significance (*p*-value) for g_1 and g_2 in cancer samples, and f is a function of two differential expression variables and one co-expression correlation variable. Then, we use Fisher's test [9] to define function f as a combination of the statistical significance of tests. Therefore, the activity of edges can be calculated by the following equation:

$$activity\left(e\left(\langle g_1, g_2\rangle\right)\right) = -2\sum_{i=1}^{k}\log\left(p_i\right), \tag{B.2}$$

where p_i is the *p*-value of individual test *i* and k is total number of tests. We set $k = 3$ and use the combined value in Equation (B.2) to check whether an edge is active. Mathematically, $activity \sim \chi^2_{2k}$ [8] and when the threshold of *p*-value is 0.05, the threshold of activity is 12.59. Thus, we define one edge as active for one cancer in the

human signaling network only if its activity is more than 12.59; that is, we reserve the active edges of each cancer in the human signaling network and we consider the edge activity as original edge weight $w(e)$. Additionally, somatic mutation is a strong signal in cancers, which should be taken into account in the process of constructing a cancer-related activated signaling network. To avoid eliminating genes with lower mutation frequency that are closely related to cancers, we consider all genes with a mutation frequency larger than zero to be somatically mutated.

Broadly, the process of constructing a cancer-related activated signaling network can be summarized as three steps: First, we construct the human signaling network as the background network. Second, we obtain active edges through the expression data of control and disease samples for each cancer. Finally, we screen genes using their mutation frequency. Each cancer network is one layer in the 16-layer cancer-related signaling network.

Specifically, in the cancer-related signaling network, the weight of edge *e* in OINs can be described as $\hat{w}(e)=\sum_{l=1}^{L} \mathrm{I}_{e\in\left(E_l^o \cup E_l^c\right)}(e)\times 10^{1+\lg|E_l|}+rank\left(w(e),E_l\right)$, where $|E_l|$ is the number of edges in G_l; and $rank\left(w(e),E_l\right)$ denotes the rank of edge *e* among all edges in E_l, which ranges from 1 to $|E_l|$. The larger the weight of edge e ($w(e)$), the higher the assigned rank of edge e. Correspondingly, edges with larger activity are detected among edges with equal CVs because we ensure that the first term of $\hat{w}(e)$ is larger than the second term $rank\left(w(e),E_l\right)$. $\hat{w}(e)$ can be changed to adapt to a practical application.

C. Pathway-based enrichment

We use ConsensusPathDB [10] to perform pathway-based enrichment analysis and select functional pathways from at most three sources of databases that contain KEGG, Reactome, and WikiPathways. We set the *p*-value cutoff to 0.01 and minimum overlap with the input list ($cp\in$CCPs of one cancer) to 2.

D. Drug repositioning

D.1 Conditions of drug repositioning with CCPs for BCLLS

From the analysis in the main text, the drug targets are inclined to be located upstream of the CCPs; therefore, we mainly focus on long CCP in CCPs. Before drug repositioning, we delete the CCPs that are constituted by only one edge. We obtain all FDA-approved drugs, called FA-BCLLS, which can remedy any of the cancers in BCLLS from the repoDB [11]. Then, we retain all drug targets *T* from DrugBank[12] for each drug in FA-BCLLS. We represent the CCPs for BCLLS as $CCPs_1 - CCPs_5$

and genes in the CCPs of one cancer as CCP genes. For every target t ($t \in T$), if it is in the CCP genes of one cancer c ($c \in$BCLLS) and at least one FDA-approved drug d contains target t can remedy cancer c in repoDB, we predict that drug d can remedy cancer c, and the correlation of drug d and cancer c is called a known *d-c* pair. We conduct drug repositioning based on the known *d-c* pair, that is, we predict that drug d can remedy another cancer in BCLLS for which there is no known *d-c* pair, but target t is in the CCP genes of the cancer. Thus, we can obtain all *d-c* pairs we predict based on targets T and CCPs.

Additionally, we consider three conditions for drug repositioning with the CCPs and further filter some *d-c* pairs. First, we only retain drug target t ($t \in T$) when it appears in the CCP genes of at least two cancers (i.e., $\sum_{i=1}^{5} I\left(t, CCPs_i\ genes\right) \geqslant 2$. If target t occurs in $CCPs_i\ genes$, $I\left(t,\ CCPs_i\ genes\right)$ is 1; otherwise, it is 0, and there exists at least one *d-c* pair that is a known therapeutic relation (i.e., known *d-c* pair) .

Second, we only consider target t located upstream of the CCPs. We define whether target t is located upstream of the CCPs using the proportion of the position of target t to the length of $CCPs_i^t$ ($pos\text{-}prop_t$), where $CCPs_i^t\left(i \in [1,5]\right)$is a CCP that contains target t in the i-th cancer among BCLLS:

$$pos\text{-}prop_t = pos\ of\ \ t \text{ in } CCPs_i^t / length \text{ of } CCPs_i^t, \quad \text{(D.1)}$$

where pos of t in $CCPs_i^t$ is the position of t in $CCPs_i^t$. For the path A→B→C, the pos of B in $CCPs_i^t$ is 2. The length of $CCPs_i^t$ represents the number of genes in $CCPs_i^t$.

Additionally, we believe that target t is located upstream of $CCPs_i^t$ when $pos\text{-}prop_t$ satisfies

$$pos\text{-}prop_t \le \begin{cases} \lceil n/2 \rceil / n & if\ \ n\ is\ odd \\ 0.5 & if\ \ n\ is\ even \end{cases} \quad \text{(D.2)}$$

where n is the length of $CCPs_i^t$.

Third, we consider the overlap between the CCPs containing target t:

$$overlap_{prop} = \left(CCPs_i^t \cap CCPs_j^t\right) / \left|CCPs_i^t\right|. \quad \text{(D.3)}$$

For the i-th cancer among BCLLS, there exists a known d-(i-th) cancer pair based on target t, whereas the d-(j-th) cancer pair is unknown for the j-th cancer. Thus, we consider that target t satisfies $overlap_{prop} \ge 0.5$. Particularly, we only retain *d-c* pairs obtained by drug repositioning with the CCPs based on target t only when one target $t \in T$ and t is one target of d that satisfies all three conditions mentioned above.

D.2 Drug repositioning with disease genes and disease modules

We consider disease genes or modules of a cancer as the CCP genes of the cancer. We predict that one drug *d* in FA-BCLLS can remedy cancer *c* in BCLLS if target *t* (*t* is one target of *d*) is in the CCP genes of cancer *c*. For the disease genes and C3 modules of BCLLS, no target satisfies the first condition mentioned above, whereas targets exist that satisfy the first condition for DIAMOnD modules. Therefore, for DIAMOnD modules, we only consider *d-c* pairs to be true if drug target $t \in T$ (t is one target of *d*) satisfies the first condition.

D.3 Human Protein-Protein Interactome (PPI)

The human PPI was compiled by Cheng et al. [13,14], who collected multiple interactions that included binary interactions, literature-curated interactions, signaling interactions, and kinase-substrate pairs. We obtain the human interactome network that includes 239,305 interactions among 16,461 unique proteins.

D.4 Drug repositioning using the proximity method

We obtain the upstream genes from each CCP of the CCPs in each cancer among BCLLS. The definition of upstream genes varies from the former 10% to the former 50% of genes of the CCPs. Then, we acquire the C3 module with the upstream genes of each cancer. We use the proximity method [15] to measure the distance between drugs in DrugBank and the C3 module of disease genes or upstream genes from CCPs in the PPI network for each cancer. We consider the known drug targets of each drug in DrugBank as *T* and disease genes in the C3 module as *S*. The distance between drug *T* and disease module *S* is evaluated as

$$d = \left(1/|T|\right) \sum_{t \in T} \min_{s \in S} d\left(s,t\right). \quad \text{(D.4)}$$

Comparing the distance with random degree-preserving randomization, we capture the statistical significance (z-score, $z = \dfrac{d-\mu}{\sigma}$) to measure how far drug *T* is from disease module *S*. Additionally, we rank the drugs using the z-score. Then, we consider FDA-approved drugs from repoDB as validation data and use all predicted drugs with an unlimited z-score or drugs with a z-score less than 0 to compute the ROC curves and AUC scores.

Supplementary Reference

1. Gómez-Gardeñes J, Moreno Y. 2006 From scale-free to Erdos-Rényi networks. *Phys. Rev. E - Stat. Nonlinear, Soft Matter Phys.* **73**, 1–7. (doi:10.1103/PhysRevE.73.056124)
2. In press. random—Python 3.6 documentation. See https://docs.python.org/3.6/library/random.html (accessed on 18 December 2019).
3. Cerami EG, Gross BE, Demir E, Rodchenkov I, Babur Ö, Anwar N, Schultz N, Bader GD, Sander C. 2011 Pathway Commons, a web resource for biological pathway data. *Nucleic Acids Res.* **39**, 685–690. (doi:10.1093/nar/gkq1039)
4. Tomczak K, Czerwińska P, Wiznerowicz M. 2015 The Cancer Genome Atlas (TCGA): An immeasurable source of knowledge. *Wspolczesna Onkol.* **1A**, A68–A77.

(doi:10.5114/wo.2014.47136)

5. Leek JT, Monsen E, Dabney AR, Storey JD. 2006 EDGE: Extraction and analysis of differential gene expression. *Bioinformatics* **22**, 507–508. (doi:10.1093/bioinformatics/btk005)
6. Stuart JM, Segal E, Koller D, Kim SK. 2003 A Gene-Coexpression Network for Global Discovery of Conserved Genetic Modules. *October* **302**, 249–255.
7. J. R, A.K. D, W. Z. 2010 A general co-expression network-based approach to gene expression analysis: Comparison and applications. *BMC Syst. Biol.* **4**.
8. Liu ZP, Wang Y, Zhang XS, Xia W, Chen L. 2011 Detecting and analyzing differentially activated pathways in brain regions of Alzheimer's disease patients. *Mol. Biosyst.* **7**, 1441–1452. (doi:10.1039/c0mb00325e)
9. Mosteller F, Fisher RA. 1948 Questions and Answers. *Am. Stat.* **2**, 30–31.
10. Kamburov A, Stelzl U, Lehrach H, Herwig R. 2013 The ConsensusPathDB interaction database: 2013 Update. *Nucleic Acids Res.* **41**. (doi:10.1093/nar/gks1055)
11. Brown AS, Patel CJ, Characteristic S. 2017 Data Descriptor: A standard database for drug repositioning. *Sci. Data* , 1–7. (doi:10.1038/sdata.2017.29)
12. Wishart DS *et al.* 2018 DrugBank 5.0: A major update to the DrugBank database for 2018. *Nucleic Acids Res.* **46**, D1074–D1082. (doi:10.1093/nar/gkx1037)
13. Zhou Y, Hou Y, Shen J, Huang Y, Martin W, Cheng F. 2020 Network-based drug repurposing for novel coronavirus 2019-nCoV/SARS-CoV-2. *Cell Discov.* **6**. (doi:10.1038/s41421-020-0153-3)
14. Cheng F, Kovács IA, Barabási AL. 2019 Network-based prediction of drug combinations. *Nat. Commun.* **10**, 1197. (doi:10.1038/s41467-019-09186-x)
15. Guney E, Menche J, Vidal M, Barábasi AL. 2016 Network-based in silico drug efficacy screening. *Nat. Commun.* **7**, 1–13. (doi:10.1038/ncomms10331)
